# Protocol for implementation of an evidence based parentally administered intervention for preterm infants

**DOI:** 10.1186/s12887-021-02596-1

**Published:** 2021-03-24

**Authors:** Rosemary White-Traut, Debra Brandon, Karen Kavanaugh, Karen Gralton, Wei Pan, Evan R. Myers, Bree Andrews, Michael Msall, Kathleen F. Norr

**Affiliations:** 1 Department of Nursing Research and Evidence-Based Practice, Children’s Wisconsin, Milwaukee, WI USA; 2 Women, Children and Family Health Science, College of Nursing, University of Illinois at Chicago, Chicago, IL USA; 3 School of Nursing, Duke University, Durham, North Carolina USA; 4 School of Nursing and Department of Population Health Sciences, Duke University School of Medicine, Durham, North Carolina USA; 5 Division of Women’s Community and Population Health, Department of Obstetrics & Gynecology, Duke University School of Medicine, Duke University, Durham, North Carolina USA; 6 College of Medicine, Department of Pediatrics, University of Chicago, Chicago, IL USA

**Keywords:** Preterm infant, Behavioral intervention, Parent engagement, NICU implementation, CFIR

## Abstract

**Background:**

Multi-sensory behavioral interventions for preterm infants have the potential to accelerate feeding, growth, and optimize developmental trajectories and increase parents’ interactive engagement with their infants. However, few neonatal intensive care units (NICUs) provide evidence-based standardized early behavioral interventions as routine care. Lack of implementation is a major gap between research and clinical practice. H-HOPE, is a standardized behavioral intervention with an infant- directed component (Massage+) and a parent-directed component (four participatory guidance sessions that focus on preterm infants’ behaviors and appropriate responses). H-HOPE has well documented efficacy. The purpose of this implementation study is to establish H-HOPE as the standard of care in 5 NICUs.

**Methods:**

The study employs a Type 3 Hybrid design to simultaneously examine the implementation process and effectiveness in five NICUs. To stagger implementation across the clinical sites, we use an incomplete stepped wedge design. The five participating NICUs were purposively selected to represent different acuity levels, number of beds, locations and populations served. Our implementation strategy integrates our experience conducting H-HOPE and a well-established implementation model, the Consolidated Framework for Implementation Research (CFIR). The CFIR identifies influences (facilitators and barriers) that affect successful implementation within five domains: intervention characteristics, outer setting (the hospital and external events and stakeholders), inner setting (NICU), implementers’ individual characteristics, and the implementation process. NICUs will use the CFIR process, which includes three phases: Planning and Engaging, Executing, and Reflecting and Evaluating. Because sustaining is a critical goal of implementation, we modify the CFIR implementation process by adding a final phase of Sustaining.

**Discussion:**

This study builds on the CFIR, adding Sustaining H-HOPE to observe what happens when sites begin to maintain implementation without outside support, and extends its use to the NICU acute care setting. Our mixed methods analysis systematically identifies key facilitators and barriers of implementation success and effectiveness across the five domains of the CFIR. Long term benefits have not yet been studied but may include substantial health and developmental outcomes for infants, more optimal parent-child relationships, reduced stress and costs for families, and substantial indirect societal benefits including reduced health care and special education costs.

**Trial registration:**

ClinicalTrials.gov registration number NCT04555590, Registered on 8/19/2020.

## Background

### The high cost of preterm birth

Premature birth is associated with immaturity of major organs and regulatory behaviors, feeding difficulties, and slower early growth, leading to longer initial hospitalization and increased economic costs [1–14]. Parents of preterm infants also experience an increase in stress, anxiety, depressive symptoms, and lack of confidence about parenting, which also leads to less optimal parent-infant interaction and adversely impacts infant growth and development [15–25]. Preterm births account for only 10–12% of births in the US, but the cost of preterm birth accounts for more than 40% of the total costs of birth hospitalizations [1, 5, 7, 26–28]. Post-discharge, 19% of preterm infants experience ongoing medical conditions, increasing average outpatient costs in the first year to approximately $10,000, and prescription costs to approximately $1800 [27, 29]. The first year total health care costs for preterm infants are three times higher when compared to full-term infants, reaching $14 billion in 2017 [30]. For very preterm infant survivors (28–31 weeks gestation), and extremely preterm infant survivors (< 28 weeks gestation), these medical costs are even higher.

Early developmental interventions have the potential to improve outcomes for preterm infants and families. Importantly, early investment in preterm infant development has high potential impact on infant short and long term outcomes, parent-infant relationships and public health and education costs [5, 7, 27, 31]. The Physical Environment Exploratory Group endorses use of sensory interventions, including massage, as standard care after 30–31 weeks post-menstrual age (PMA, calculated as gestational age at birth plus chronological age) [32]. A large body of evidence supports that these interventions accelerate early brain development and maturation of visual function, improve feeding ability and growth factors, as well as health, long-term language, motor, and cognitive development. For the parents, the interventions increase interactive engagement with their infants and parent-infant interaction, which may mitigate the effects of increased parental stress [18, 32–48]. Multi-sensory developmental interventions that have a behavioral focus have been found especially effective in accelerating feeding, growth, and development [32, 33, 43, 49, 50]. However, few neonatal intensive care units (NICUs) provide evidence-based standardized parent directed early developmental interventions that have a behavioral focus as routine care. Lack of implementation is a major gap between research and clinical practice.

H-HOPE is a standardized intervention ready for dissemination. Our team developed the H-HOPE intervention (Hospital to Home: Optimizing the Preterm Infant’s Environment) to promote early infant development and parental engagement. H-HOPE consists of two components, one infant-directed and one parent-directed. Together, these components simultaneously address the needs of both preterm infants and their parents, especially their need for mutual engagement in NICUs. Our early research documented the efficacy of H-HOPE’s infant-directed component, the ATVV, which provides **A**uditory (voice), **T**actile (moderate touch massage), **V**isual (eye to eye), and **V**estibular (rocking) stimulation. We renamed the ATVV as Massage+ to be more readily understood by parents. Massage+ is initiated when infants are ready for social interaction around 31–32 weeks. When tested with infants at varying degrees of risk, Massage+ improved infant oral feeding and social interactive skills prior to and during feeding, improved their capacity to attain alert behavioral states more optimal for feeding and parent-infant interaction, in-hospital growth (weight gain) and development, and reduced length of hospital stay [11–14, 51–55]. However, Massage+ alone did not address parents’ reported distress and their need for participatory guidance and social support to engage with their infants, despite the infants’ immature behaviors. To meet parents’ needs, we developed the parent-directed component of H-HOPE, which consists of 4 participatory guidance sessions for parents, 2 during the NICU stay and 2 during the transition to home.

Together, H-HOPE’s components optimize early infant behavior and parental capacity to engage in social interaction. In previous research, we found improved developmental maturation (more mature behavioral states, increased frequency of orally directed behaviors, faster transition from gavage to oral feeding, improved sucking organization and motor development), greater in-hospital growth (weight gain and length), and enhanced engagement, social interaction and infant responsivity [43, 49, 50, 56]. Additionally, H-HOPE reduced initial in-hospital costs (net savings of $13,976 per infant after adjusting for the mean intervention cost of $680) [57] and health care use from discharge through 6-weeks corrected age (CA, chronological age corrected for weeks born preterm) [58]. With established efficacy and a manualized standardized protocol, H-HOPE is ready for widespread implementation, making H-HOPE uniquely suited as an early behavioral intervention for preterm infants in NICUs.

### Preparing NICUs to adapt H-HOPE as the standard of care

To reduce the gap between research and implementation, clinicians need a strategy to introduce an early behavioral intervention involving parents within the high-acuity NICU. Our implementation strategy integrates our experience conducting H-HOPE and a well-established implementation model, the Consolidated Framework for Implementation Research (CFIR) [59]. The CFIR identifies influences (facilitators and barriers) that affect successful implementation within five domains: intervention characteristics, outer setting (the hospital and external events and stakeholders), inner setting (NICU), implementers’ individual characteristics, and the implementation process. We selected the CFIR because the framework integrates the best features of previous models [59] and captures the complexity of real-world implementation to identify site-specific factors and patterns across cases [60, 61]. The framework has been used for diverse interventions in inpatient, clinic and community settings. A systematic review in 2016 identified 26 empirical studies using the CFIR, [62] and there are numerous studies published since then [43, 63–71]. However, only two previous studies occurred in a high acuity inpatient setting (adult acute care units) [63, 70] and no prior studies include the NICU setting. The CFIR provides a systematic approach that will be used to guide the process of implementing H-HOPE in NICUs, to recognize facilitators of successful implementation, and to identify and address implementation barriers across different sites. NICUs will use the CFIR process, which includes three phases: Planning and Engaging, Executing, and Reflecting and Evaluating. We modified the CFIR implementation process by adding a final phase of sustaining H-HOPE without continued support from the research team, because sustaining is a critical goal of implementation [72, 73].

In addition to adopting an implementation model, we modified the H-HOPE protocol used in the efficacy study to further facilitate implementation of H-HOPE as the standard of care for this initial study, nationwide and eventually globally. We expanded infant and parent eligibility criteria to reach a wider range of preterm infants and their parents who can benefit from an early behavioral intervention. In collaboration with Pathways.org, an organization dedicated to early childhood development, we developed a web-based support package of training webinars, Massage+ videos and downloadable materials for H-HOPE providers and trainers (www.Pathways.org). We replaced costly post-discharge home visits with screen time (e.g. FaceTime). Through regular interactive consultation at each site, the research team will share their experiences to support implementation and lessons learned across sites will be incorporated into the web-based support package. Because every NICU represents a different context, we view the implementation process as a collaboration that balances fidelity to intervention core components to maintain H-HOPE’s effectiveness, while adapting non-essential aspects (e.g. training format, who provides services) to fit each site’s unique set of needs [74, 75].

### Study aims

#### Aim 1 (implementation success)

Identify the degree of implementation success at each clinical site every 2 months from Executing through 6 months after supported implementation ends (*primary implementation outcome)*. Success measures include: Sustainability (offering H-HOPE), Reach (% of eligible parent-infant units receiving H-HOPE) and Degree of Implementation (mean H-HOPE services received).

#### Aim 2 (effectiveness)

Evaluate the effectiveness of H-HOPE compared to a pre-H-HOPE comparison cohort. We hypothesize that the H-HOPE cohort will have:

H2a. More positive infant outcomes including improved growth in the NICU and fewer illness visits from initial discharge through 6-weeks post discharge *(primary H-HOPE effectiveness outcomes)*.

H2b. Lower initial hospitalization costs from entry into H-HOPE through discharge, taking into account the implementation costs of the H-HOPE program per infant, resulting in long-term hospital/health system cost savings *(secondary H-HOPE effectiveness outcome).*

H2c. More positive parent outcomes, including increased visits in the NICU, lower NICU- related worry, lower anxiety and depression, and greater confidence in premature infant care *(exploratory H-HOPE effectiveness outcomes)*.

#### Aim 3 (influences associated with success and effectiveness)

Determine the influences of the 5 CFIR domains associated with success (Aim 1) and the primary H-HOPE effectiveness infant outcomes (Aim 2).

## Methods/design

**Fig. 1:**
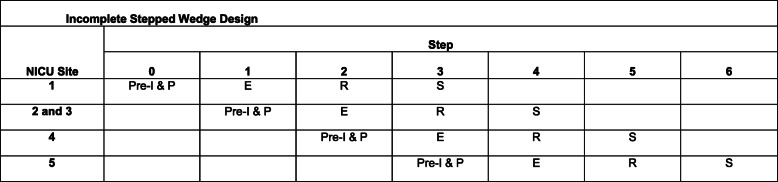
Incomplete Stepped Wedge Design. Pre-I & P = 6 months of pre-implementation plus Planning and Engaging during which Pre-H-HOPE Comparison Cohort Data are collected simultaneously, E = 6 months of Executing at the end of which posttest will be taken, R = 2 months of Reflecting and Evaluating, S = 6 months of Sustaining. The order of implementation was randomly assigned

The study employs a Type 3 Hybrid design [76] to simultaneously examine the implementation process and effectiveness in five NICUs. To stagger implementation across the clinical sites, we use an incomplete stepped wedge design [77] (see Fig. 1). This design is more advantageous than conventional cluster randomized trials because it requires fewer NICUs (clusters), allows all NICUs to implement H-HOPE, and permits an estimate of treatment effects both within- and between-NICUs [77–80]. To eliminate systematic bias due to order of initiation, start dates were determined by a random number generator. Each NICU implements H-HOPE in 3 phases: Planning and Engaging (6 months), Executing (6 months), and Reflecting and Evaluating (2 months) [81, 82]. Implementation is followed by 6 months of Sustaining.

To evaluate effectiveness, we chose not to use a randomized design with simultaneous intervention and control comparison groups because it would be confusing for nursery staff and parents to have two different standards of care. Because this is an implementation study, H-HOPE will be introduced as the standard of care. All eligible infants will receive H-HOPE whether or not their parents enroll in the research study. Instead, we compare parent and infant outcomes before and after the introduction of H-HOPE at each site.

### Study setting and sample

To maximize generalizability of results, we selected 5 NICUs that are diverse in size (26–72 beds), acuity level (one level II, two level III, and two level IV NICUs), location (urban/suburban/rural), and population served (See Table 1). All participants will give written informed consent. Our study population is anticipated to include 44% White, 26.6% Black/African American, 0.6% Native American, 6% Asian, 19% Latinx, and 3.7% other. These estimates include substantially fewer Latinx, a group with the lowest prematurity rate of 9.9%, and larger proportion of Blacks and Native Americans, the groups with the highest prematurity rates in the US (Blacks, 13.8%; Native Americans, 11.4%) [83]. All of the NICUs provide developmental care that includes non-disruption of sleep cycles and skin-to-skin care when infants are stable. Comparisons across units will help identify NICU characteristics that make it more challenging to implement and sustain H-Hope. Sites 2 and 3 are in the same health system. Therefore, these NICUs will implement H-HOPE simultaneously and are considered as one site for effectiveness evaluation (Aim 2). However, they will be analyzed as separate sites for success and influences (Aims 1 and 3) because the inner settings differ, e.g., they have different nursing staff and hospital administrators.

**Table 1:** Expected H-HOPE Cohort and Site Characteristics

NICU Site^a^	NICU Level	Annual Admissions^b^	# Eligible in 6 Months^c^	# Enrolled^c^	# Retained^c^	Race/ethnicity^d^%					% Medicaid	Location
						Wh	Blk	Am Ind	Asian	Latinx		
Site 1	III/IV	184	62	44	31	16	71.2	0	0	12.8	74.5	Urban
Sites 2 and 3	III/IV, II	454 119	222 30	155 21	108 15	38	48	8	2	4	70	Rural/ Suburban
Site 4	III	297	148	111	77	62.8	6	0	15.5	14.9	40	Suburban
Site 5	III	150	69	48	34	89	1.8	3	2.2	4	36	Rural
TOTAL(s)		1205	542	379	265	42.7	34.9	4.5	4.8	7		

^a^In order of Random Assignment; ^b^Annual Admissions for 23–35 wk. GA; ^c^# eligible, # enrolled is at a 70% enrollment rate & # retained at 6-weeks post discharge for H-HOPE Cohort; ^d^White, Black/African American, American Indian, Asian, Latinx

### Sample

Aim 1 (Implementation success) data will be obtained from each site’s de-identified aggregated electronic health record (EHR) data.

Aim 2 (Effectiveness) participants include both parents and infants from two cohorts of subjects: the Pre-H-HOPE Comparison Cohort and the H-HOPE Cohort. The Pre-H-HOPE Comparison Cohort consists of two sub-cohorts: 1) Consented Pre-H-HOPE Parent-Infant Units, for whom all parent and infant data are collected and 2) Non-Consented Pre-H-HOPE Infants for whom only de-identified existing infant growth and cost data are used. The non-consented Pre-HOPE Cohort is added to have a large enough sample for propensity score matching to test H-HOPE’s impact on infant growth and initial hospitalization costs.

#### Eligibility criteria and recruitment

For H-HOPE and Pre-H-HOPE Consented parent-infant dyads, infants are eligible if they are born at 23–35 weeks gestational age, at least 31–32 weeks PMA, and clinically stable. Parents are mothers, fathers, or other significant family member who intend to act in the role of a parent, regardless of biological relationship to the infant, gender identity or sexual orientation. In most cases the biological mother is identified as the primary parent and she will be asked to name the second parent. The Non-Consented Pre-H-HOPE Infants must meet the same infant criteria, however no parents are involved.

#### Sample size and power for aim 2 effectiveness

Based on participating sites’ data, 542 infants will be eligible for the H-HOPE cohort during the 6-month Executing phase. Of the available infants, we expect to enroll at least 70% (*n* = 379 parent-infant pairs) and to retain 265 through 6-weeks following the infant’s discharge. During a 12-month period before H-HOPE implementation, 1084 eligible infants will be available for the Pre-H-HOPE Comparison Cohort in two sub-cohorts.

The first sub-cohort, the Consented Pre-H-HOPE Parent-Infant Units, is recruited at the same time as the Planning and Engaging phase at each site and will contribute to all outcome analyses (*n* = 542). The retention rates will be the same in the Consented Pre-H-HOPE sub-cohort and the H-HOPE cohort (*n* = 265). The second sub-cohort, the Non-Consented Pre-H-HOPE Infants (*n* = 542), will contribute only retrospective infant growth and cost data obtained from de-identified EHR and cost records in the 6 months prior to the start of Planning and Engaging. Based on our prior research, near medium to large effect sizes are expected for the primary outcomes of infant growth and health care utilization following discharge [49, 57] and the secondary hospital cost outcome [58]. Using the STATA package *stepped wedge* [84], the power analysis found that there will be at least 80% of power to detect a medium effect of Cohen’s *d* = − 0.50 at a significance level of α = .05 in this stepped wedge design with 4 sites and an average of at least 50 subjects per site (or 200 in total) across 4 staggered time points, assuming the intra-class correlation coefficient (ICC) is .1, a common ICC found in health and behavioral research [85, 86]. Assuming ICC as .05, which is common ICC health cost analysis [87–89], research would require at least 30 subjects per site (or 120 in total). Thus, in this proposed study, the anticipated total sample size of 265 will be adequate to detect a medium effect for all outcome measures.

The Aim 3 sample includes hospital personnel and parents receiving H-HOPE at each site. Consented H-HOPE parents will be randomly selected (15 parents per site, 75 total) to be interviewed prior to discharge and when their infant is 6-weeks post discharge. Purposively selected hospital personnel include at least one hospital administrator, NICU leadership (e.g. medical director and nurse manager), clinical site H-HOPE team members (2–4 per site), and selected staff who work in the NICU (10 per site) who have delivered H-HOPE, especially champions and non-supporters. Each of these selected hospital personnel will participate in 3 short qualitative interviews about their experience with H-HOPE. In addition, all registered staff nurses who work at least 50% in the NICU will complete a brief survey regarding engagement and burnout during the three project phases.

### Implementation study conditions

Infants and parents in the Pre-H-HOPE Comparison Cohort receive usual NICU care and parent interactions with NICU staff.

The H-HOPE Cohort of infants and parents receive H-HOPE in-hospital and during the transition to home. Four parent-directed participatory guidance sessions offer social support to parents and help them learn the nuances of preterm infant behavior and how to interact with their infant and provide Massage+. The initial in-hospital session occurs when H-HOPE begins. Preparing for the transition to home begins during the second in-hospital session shortly before discharge, and continues with sessions at 2–3 days and 7–15 days post-discharge via screen time. The infant-directed multi-sensory component of H-HOPE Massage+, is administered for 15 min prior to a feeding twice daily, starting when the infant is physiologically stable and ready for social interaction (≈ 31–32 weeks PMA for infants < 30 GA at birth). Twice daily administration was selected based on our previous research [11, 12, 14, 43, 50, 52–54, 56, 90–92]. Ideally, parents would always administer Massage+, however few parents are able to visit twice daily. Therefore, trained staff will provide the intervention in the parents’ absence. Parents will continue the Massage+ at home twice daily until 1-month CA. In our previous research with low income minority parents, 95% continued Massage+ at home [43, 52].

To assess H-HOPE fidelity during Executing at each site, we document completeness and quality of services provided for each parent-infant unit, as recommended in recent studies [93, 94]. Completeness of services in-hospital and during the transition to home is recorded in the EHR and assessed as part of Aim 1. The quality of H-HOPE services is examined periodically using standardized forms by site staff to ensure that fidelity at that site is maintained over time.

The H-HOPE Implementation Support Package consists of 2 components. A multimedia web-based program provides materials and training which are available to parents and staff on the Pathways.org website (www.Pathways.org). Interactive assistance from the investigative team to each site provides NICU-specific consultation throughout the 3 phases of implementing H-HOPE. This consultation has been shown to facilitate implementation [82, 95]. On line webinars will be supplemented by in person training and support at the infant’s bedside, especially when the staff administer H-HOPE for the first time. Each site will have several master trainers. A program for master trainers will be developed using examples from the first two sites trainings.

### Measures

#### Aim 1 primary implementation outcome measures

We examine three measures of success recommended for late-phase implementation by Proctor et al. [72]. Sustainability measures whether the site is providing H-HOPE. Reach measures the proportion of eligible parent-infant units who receive H-HOPE at that site. Degree of Implementation measures the mean H-HOPE services received per parent-infant unit. The research team will consult with each site to build within the EHR infant eligibility for H-HOPE, documentation of Massage+ and the parent participatory guidance sessions. These success measures will be obtained from each site’s EHR which will record eligibility and receipt of H-HOPE parent and infant services. If the site chooses to discontinue H-HOPE, we will conduct the final interview and end evaluation.

#### Aim 2 effectiveness outcome measures

Our primary effectiveness outcome measures include infant growth from birth through hospital discharge and acute care visits from initial discharge through 6-weeks post discharge. Our secondary outcome is mean hospitalization cost per day from entry into H-HOPE until discharge adjusted for the per-infant cost of providing H-HOPE. Our exploratory outcome includes 3 parent measures (See Table 2).

**Table 2:** Aim 2 Effectiveness Outcome Measures

Outcome Variable	Operational Measure	Timing			Data Source
		D/C	2-wks	6-wks	
Infant (Primary)	1. Growth-weight and length;	●			EHR Parent report
	2. Fewer acute care visits discharge to 6-weeks post discharge		●	●	
Costs (Secondary)	3. Average hospitalization cost/day (direct costs) from eligibility for H-HOPE through discharge, adjusted for the H-HOPE cohort only by adding the cost per infant to implement H-HOPE* (training, coordinating, time to deliver H-HOPE and to arrange screen time visits)	●			Hospital cost data & H-HOPE records
Costs (Secondary)	4. # Parent NICU visits/days of hospitalization (each parent)	●			EHR Parent Survey
	5. Parental Worry [96]	●		●	
	6. Karitane Parenting Confidence Scale [97]	●		●	
	7. Parent depressive symptoms, PCORI short form [98]	●		●	
	8. Parental Worry [96]	●		●	

D/C=Discharge; 2-wks = 2-weeks post D/C; 6-wks = 6-weeks post discharge; EHR = Electronic Health Record; ^*^H-HOPE Planning, Training and Delivery In-hospital and with Transition to home costs

Covariates for Aim 2 analyses include infant sex, partial implementation of H-HOPE, and number of parent NICU visits. Other infant and parent characteristics will be included as covariates if they are related to the specific outcome variable of interest. Infant characteristics include race/ethnicity, gestational age at birth, single/multiple birth, sex, Neurobiological Risk Score (NBRS) [99, 100], and days of mechanical ventilation. Parent characteristics include primary parent education, age, income, marital status, self-identified race/ethnicity, relationship to the infant, and maternal parity.

#### Aim 3 secondary implementation outcome measures

Aim 3 uses mixed methods analyses to integrate data collected for this aim regarding influences (facilitators or barriers) in each of the 5 CFIR domains with data from Aim 1 (implementation success) and Aim 2 (effectiveness for the primary infant outcomes). Data specific for Aim 3 include: qualitative interviews with hospital personnel and parents, H-HOPE team meeting records, research team observation notes, nurse survey data, and fidelity data from H-HOPE records. Separate interview guides for hospital personnel and parents include questions about domains and constructs within the CFIR framework relevant to their experience with H-HOPE. Hospital personnel are interviewed at 3 time points (Planning and Engaging, end of Executing, and end of Sustaining). Data about individual characteristics that influence implementation come from registered staff nurse surveys conducted at the same time as the interviews. Parents are interviewed twice: close to discharge and again after discharge when the infant has been home for 6 weeks.

### Study procedures

#### Overall study coordination

Procedures for this multisite complex implementation and evaluation study, are coordinated within three Centers: the Implementation Center, the Quantitative Data Center, and the Qualitative and Mixed Methods Data Center. Each Center is guided by a co-investigator who works collaboratively to facilitate overall project coordination and management.

The Implementation Center coordinates the implementation of H-HOPE at each clinical site, working closely with the H-HOPE Clinical Site Implementation Team. Each site’s H-HOPE team is comprised of the Clinical Site Nurse, NICU leadership and other clinicians. Collaboratively with the co-investigator and the study’s Project Director, the site H-HOPE team develops the site-specific NICU protocol and plan for staff training, coordinates the implementation process in the NICU, and serves as champions who provide resources and support for staff throughout implementation.

During the Planning and Engaging phase, the Implementation Center will train the clinical site’s H-HOPE Implementation team. The H-HOPE Team in collaboration with the Center Director, will identify the necessary processes for establishing the H-HOPE program as standard of care, taking into account the NICU culture and their capability for adaptation. During the Executing phase, the NICU will implement H-HOPE according to their plan. During the Reflecting and Evaluating phase, the H-HOPE Implementation team will participate in preparing a report describing the effectiveness of the H-HOPE program on the infants and parents in their NICU, as well as hospital personnel. This report will be shared with hospital administrators and NICU leadership to provide evidence for sustaining or ending the H-HOPE program. During independent Sustaining, the H-HOPE Implementation Team can contact the Implementation Center at any time for additional consultation.

The Quantitative Data Center provides oversight and coordination for all quantitative data-related operations and conducts the quantitative data analyses for Aim 1 (degree of implementation) and Aim 2 (effectiveness). Prior to Planning and Engaging, the Center works with each site’s informatics team to modify their EHR build so the H-HOPE eligibility, initiation and specific services received can be documented directly in the EHR. The Center also oversees the REDCap database construction and management for participant screening, study enrollment, eConsenting, study monitoring, and quantitative data collection. During implementation at each site, the Center works closely with each site to obtain relevant data throughout the study, shares site results and helps prepare the report that the site administrative team will use to decide whether to continue H-HOPE as the standard of care. Each site has a H-HOPE research team that is responsible for consenting both pre-H-HOPE and H-HOPE parents, facilitating and monitoring data collection, and coordinating data collection and transfer with the data Centers.

The Qualitative and Mixed Methods Data Center manages and analyzes all qualitative and mixed methods data-related operations. The primary focus of the Center is ongoing mixed methods data analysis and interpretation to address Aim 3. The qualitative interview data are managed within the Center and analyzed as described below. The relevant quantitative data are obtained from the Quantitative Data Center. At each site, the Center works with the H-HOPE team to obtain H-HOPE team meeting records and to complete interviews with selected hospital personnel and selected H-Hope parents.

### Analysis plan

#### Aim 1 analysis

Aim 1 measures, Sustainability, Reach, and Degree of Implementation, are analyzed by the Quantitative Data Center, using descriptive statistics and visual displays.

#### Aim 2 analysis

Aim 2 evaluates the effectiveness of H-HOPE including infant growth, health care utilization following discharge, infant hospital costs. Aim 2 also explores the impact of H-HOPE on parent outcomes detailed in Table 2*(exploratory outcomes)*. The Quantitative Data Center analyzes Aim 2 measures.

Before analytic modeling, propensity score methods [101–104] will be utilized to reduce any selection bias by ensuring balanced distributions of infant and primary parent characteristics between Pre-H-HOPE and H-HOPE infants. For the growth and infant hospital costs outcomes, propensity scores will first be computed using available infant and parent characteristics. Then, pairs of infants will be created by matching each infant in the H-HOPE cohort with an infant in the pre-H-HOPE cohort with the same or most similar propensity scores. The final analytical models will be applied directly to propensity-score-matched pairs.

For the other Aim 2 outcomes, including health care utilization from discharge through 6-weeks post discharge and the exploratory parent outcomes, data are collected directly from parents and are not available in hospital records. Therefore, propensity score weighting will be used. In propensity score weighting, propensity scores will first be computed similarly to propensity score matching. Then propensity scores will be used to calculate inverse probability of treatment weights as weighting scores, rather than matching samples (i.e., infants or parents). The propensity weighting scores will be incorporated in weighted final analytical models preserving the entire sample for final analytical modeling.

In all the final analytical modeling, linear mixed models will be utilized to analyze normally distributed outcomes [80, 105] (e.g., infant growth and parent outcomes). Linear mixed models are an extension of linear regression models to allow estimating both between-site and within-site effects when there is a nested data structure (e.g., individual patients/infants are nested within sites). Generalized linear mixed models, an extension of linear mixed models, will be used to analyze non-normally distributed outcome measures (e.g., infant health care utilization, hospital costs).

In order for new hospitals or health systems to make informed decisions about implementation, we will estimate the incremental costs of implementing H-HOPE using a Markov state-transition model over a 5-year time horizon from the perspective of an individual hospital or health system, using standard methods [57, 106, 107]. Total implementation costs will be derived from (1) H-HOPE initial implementation costs using the observed number of hours spent for training multiplied by national estimates of hourly NICU nursing salary, (2) H-HOPE infant intervention delivery costs estimated from the propensity-weighted hospitalization costs, and (3) costs of infant healthcare utilization (number of visits multiplied by the average cost of each type of visit). In subsequent years, the costs of ongoing training will be reduced based on estimates of the number of new staff needing training. Both probabilistic and deterministic analyses will address the effect of uncertainty in cost and effectiveness estimates on the decision to implement H-HOPE, including (a) identifying the plausible values for effectiveness where H-HOPE does not result in net cost-savings over a 5 year time horizon, and (b) if cost-savings are reached within 5 years, the time for achieving cost-neutrality. Additional sensitivity analyses will examine the effects of varying assumptions about NICU admission volume and staff turnover.

#### Aim 3 analyses

Aim 3 analysis will determine influences (facilitators and barriers) in each of the 5 CFIR domains that are associated with success (Aim 1) and H-HOPE effectiveness for our primary infant outcomes (Aim 2). We follow a series of tasks and create within and across case matrices to integrate qualitative and quantitative data using well-established mixed methods analyses [108]. We first prepare site-specific case summaries using directed content analysis [109] to code the key influences on implementation success from the qualitative data to create a narrative summary and within-site matrix for each site across time. Next we examine the quantitative measures from the nurse survey and H-HOPE fidelity data. These data will be pulled into the mixed methods data analysis software, where summary statistics such as mean scores can be calculated and categorized as interval level measures (e.g., high/medium/low), and then added to the case summary. Next we identify those influences linked with success and effectiveness for each site and across all sites.

We then create interval level summary categories based on the quantitative data from Aim 1 and Aim 2 and add the levels of success and effectiveness to each site’s case summary and within site matrix. We combine these partial pictures into a single within-case summary matrix for each NICU. Finally, we determine common influences across sites associated with both success and effectiveness. We will display our findings in a single across-site matrix showing influences associated with implementation success and degree of effectiveness as well as factors affecting only one of these.

### Impact of the Covid-19 pandemic on this study

Since this implementation research was funded, the COVID-19 pandemic has drastically altered staff and parent interactions with infants in the NICU. Currently, most sites require both staff and parents to wear masks and parent visiting has been limited. These restrictions may directly impact the effectiveness of H-HOPE as Massage+ emphasizes face-to-face contact between the infant and parent or other caregiver. We plan to document specific COVID-19 related changes at each NICU during the course of this study, and we are exploring strategies such as transparent masks to reduce the disruption of COVID-19 restrictions. We will also incorporate the impact of masks and visitation restrictions on parent-infant interactions during H-HOPE into our analyses.

### Study status

At the time this manuscript was submitted for publication, the research was in the initial start-up phase. The implementation process at specific sites has not yet started and no subjects have been enrolled.

## Discussion

This study builds on one of the most widely used and systematic implementation frameworks, the CFIR, and extends its use to the NICU acute care setting. We adapted the 3-phase CFIR implementation model by adding Sustaining to explicitly observe what happens when sites begin to maintain implementation without outside support. Our mixed methods analysis systematically identifies key facilitators and barriers of implementation success and effectiveness across the five domains of the CFIR. The five participating NICUs were purposively selected to represent different acuity levels, number of beds, locations and populations served. This comprehensive approach allows us to examine both the final level of implementation success and different patterns by site over time.

Results of our analysis will identify common and site-specific implementation facilitators and barriers as well as ways sites overcame barriers. Findings will be widely disseminated to guide future expansion of H-HOPE as the standard of care. Study results will make major contributions to implementation science, especially for the introduction of complex evidence-based programs into the challenging high-acuity environment of the NICU. If the five sites in this study are successful in sustaining the program, H-HOPE will be ready for widespread adoption nationwide.

Early behavioral interventions involving parents can engage families and improve outcomes for preterm infants and their families. Although now recommended as the standard of care, this type of early intervention is rarely provided outside of research. H-HOPE brings a unique approach to early behavioral intervention by simultaneously addressing the needs of infant and parents. H-HOPE is the only evidence-based and standardized early behavioral intervention that is manualized and ready for widespread adoption. H-HOPE has already been shown to support early infant growth and development and parent-infant engagement. Long term benefits have not yet been studied yet may include short term health and developmental outcome for infants, more optimal parent-child relationships, reduced stress and costs for families, and substantial indirect societal benefits including reduced health care costs and need for special education services. This implementation study will provide a systematic strategy to scale-up H-HOPE as the standard of care in the complex high acuity NICU setting. If the 5 NICUs in this study succeed in implementing and sustaining H-HOPE, what is learned from this study will support other NICUs to adopt it. Our team will publish standardized recommendations for generalizability at the conclusion of this study.

## Data Availability

Data from this human subjects research will be shared with other investigators in accordance with the guidelines set forth by the NIH Data Sharing Policy and Implementation Guidance (March 5, 2003).

## Abbreviations

NICU: Neonatal intensive care unit
H-HOPE: Hospital to Home: Optimizing the Preterm Infant’s Environment
CFIR: Consolidated Framework for Implementation Research
PMA: Post-menstrual age
ATVV: which provides **A**uditory (voice), **T**actile (moderate touch massage), **V**isual (eye to eye), and **V**estibular (rocking) stimulation
CA: Corrected age (chronological age corrected for weeks born preterm)
EHR: Electronic health record
ICC: Intra-class correlation coefficient
NBRS: Neurobiological Risk Score
